# Supplementary material for: How neocarcerand Octacid4 self-assembles with guests into irreversible noncovalent complexes and what accelerates the assembly
Source: Commun Chem. 2022 Jan 20;5:9. doi: 10.1038/s42004-022-00624-4 (PMC9814096; doi:10.1038/s42004-022-00624-4)
Supplement: Supplementary file 2 — Description of Additional Supplementary Files [file 42004_2022_624_MOESM2_ESM.pdf]

Description of Additional Supplementary Files

File Name: Data S1

Description:

Data S1. The tLEaP input file for building the neutral Octacid4 using the AMBER HC2 library and frcmod files.

File Name: Data S2

Description:

Data S2. The Cartesian coordinates of the energy-minimized Octacid4•guest in all configurations as listed in Table S1.

File Name: Data S3

Description:

Data S3. The Cartesian coordinates of the Octacid4 conformations with one or two pairs of bidendate linkers before and after the minimization.

File Name: Video S1

Description:

The side view of the *p*-xylene self-assembly with the Octacid4 possessing two nearly-orthogonal linkers at 298 K. Host and guest are in the stick and stick-and-ball models, respectively. Carbon is in orange for host and yellow for guest, respectively. Oxygen is in red for both host and guest. Hydrogen and counter ion are not displayed for clarity.

File Name: Video S2

Description:

The top view of the *p*-xylene self-assembly with the Octacid4 possessing two nearly-orthogonal linkers at 298 K. Host and guest are in the stick and stick-and-ball models, respectively. Carbon is in orange for host and yellow for guest, respectively. Oxygen is in red for both host and guest. Hydrogen and counter ion are not displayed for clarity.

File Name: Video S3

Description:

The side view of the *p*-xylene self-assembly with the Octacid4 possessing two nearly-parallel linkers at 298 K. Host and guest are in the stick and stick-and-ball models, respectively. Carbon is in orange for host and yellow for guest, respectively. Oxygen is in red for both host and guest. Hydrogen and counter ion are not displayed for clarity.

File Name: Video S4

Description:

The top view of the *p*-xylene self-assembly with the Octacid4 possessing two nearly-parallel linkers at 298K. Host and guest are in the stick and stick-and-ball models, respectively. Carbon is in orange for host and yellow for guest, respectively. Oxygen is in red for both host and guest. Hydrogen and counter ion are not displayed for clarity.

File Name: Video S5

Description:

The side view of the *p*-xylene self-assembly with the Octacid4 possessing two nearly-coplanar linkers at 298 K. Host and guest are in the stick and stick-and-ball models, respectively. Carbon is in orange for host and yellow for guest, respectively. Oxygen is in red for both host and guest. Hydrogen and counter ion are not displayed for clarity.

File Name: Video S6

Description:

The top view of the *p*-xylene self-assembly with the Octacid4 possessing two nearly-coplanar linkers at 298 K. Host and guest are in the stick and stick-and-ball models, respectively. Carbon is in orange for host and yellow for guest, respectively. Oxygen is in red for both host and guest. Hydrogen and counter ion are not displayed for clarity.

File Name: Video S7

Description:

The side view of the 1,4-dioxane self-assembly with the Octacid4 possessing two nearly-parallel linkers at 298 K. Host and guest are in the stick and stick-and-ball models, respectively. Carbon is in orange for host and yellow for guest, respectively. Oxygen is in red for both host and guest. Hydrogen and counter ion are not displayed for clarity.

File Name: Video S8

Description:

The top view of the 1,4-dioxane self-assembly with the Octacid4 possessing two nearly-parallel linkers at 298 K. Host and guest are in the stick and stick-and-ball models, respectively. Carbon is in orange for host and yellow for guest, respectively. Oxygen is in red for both host and guest. Hydrogen and counter ion are not displayed for clarity.

File Name: Video S9

Description:

The side view of the naphthalene self-assembly with the Octacid4 possessing two nearly-parallel linkers at 298 K. Host and guest are in the stick and stick-and-ball models, respectively. Carbon is in orange for host and yellow for guest, respectively. Oxygen is in red for both host and guest. Hydrogen and counter ion are not displayed for clarity.

File Name: Video S10

Description:

The top view of the naphthalene self-assembly with the Octacid4 possessing two nearly-parallel linkers at 298 K. Host and guest are in the stick and stick-and-ball models, respectively. Carbon is in orange for host and yellow for guest, respectively. Oxygen is in red for both host and guest. Hydrogen and counter ion are not displayed for clarity.

File Name: Video S11

Description:

The side view of the *p*-xylene self-assembly with the Octacid4 possessing two nearly-orthogonal linkers and one pair of bidendate linkers at 298 K. Host and guest are in the stick and stick-and-ball models, respectively. Carbon is in orange for host and yellow for guest, respectively. Oxygen and sodium are in red and light blue, respectively. Hydrogen and counter ion, except those involving bidendate coordination, are not displayed for clarity.

File Name: Video S12

Description:

The top view of the *p*-xylene self-assembly with the Octacid4 possessing two nearly-orthogonal linkers and one pair of bidendate linkers at 298 K. Host and guest are in the stick and stick-and-ball models, respectively. Carbon is in orange for host and yellow for guest, respectively. Oxygen and sodium are in red and light blue, respectively. Hydrogen and counter ion, except those involving bidendate coordination, are not displayed for clarity.

File Name: Video S13

Description:

The side view of the *p*-xylene self-assembly with the Octacid4 possessing two nearly-parallel linkers and one pair of bidendate linkers at 298 K. Host and guest are in the stick and stick- and-ball models, respectively. Carbon is in orange for host and yellow for guest, respectively. Oxygen and sodium are in red and light blue, respectively. Hydrogen and counter ion, except those involving bidendate coordination, are not displayed for clarity.

File Name: Video S14

Description:

The top view of the *p*-xylene self-assembly with the Octacid4 possessing two nearly-parallel linkers and one pair of bidendate linkers at 298 K. Host and guest are in the stick and stick- and-ball models, respectively. Carbon is in orange for host and yellow for guest, respectively. Oxygen and sodium are in red and light blue, respectively. Hydrogen and counter ion, except those involving bidendate coordination, are not displayed for clarity.

File Name: Video S15

Description:

The side view of the *p*-xylene self-assembly with the Octacid4 possessing two nearly-coplanar linkers and two pairs of bidendate linkers at 298 K. Host and guest are in the stick and stick- and-ball models, respectively. Carbon is in orange for host and yellow for guest, respectively. Oxygen and sodium are in red and light blue, respectively. Hydrogen and counter ion, except those involving bidendate coordination, are not displayed for clarity.

File Name: Video S16

Description:

The top view of the *p*-xylene self-assembly with the Octacid4 possessing two nearly-coplanar linkers and two pairs of bidendate linkers at 298 K. Host and guest are in the stick and stick- and-ball models, respectively. Carbon is in orange for host and yellow for guest, respectively. Oxygen and sodium are in red and light blue, respectively. Hydrogen and counter ion, except those involving bidendate coordination, are not displayed for clarity.

File Name: Video S17

Description:

The side view of the 1,4-dioxane self-assembly with the Octacid4 possessing two nearly-parallel linkers and one pair of bidendate linkers at 298 K. Host and guest are in the stick and stick-and-ball models, respectively. Carbon is in orange for host and yellow for guest, respectively. Oxygen and sodium are in red and light blue, respectively. Hydrogen and counter ion, except those involving bidendate coordination, are not displayed for clarity.

File Name: Video S18

Description:

The top view of the 1,4-dioxane self-assembly with the Octacid4 possessing two nearly-parallel linkers and one pair of bidendate linkers at 298 K. Host and guest are in the stick and stick-and-ball models, respectively. Carbon is in orange for host and yellow for guest, respectively. Oxygen and sodium are in red and light blue, respectively. Hydrogen and counter ion, except those involving bidendate coordination, are not displayed for clarity.
