# Supplementary material for: How neocarcerand Octacid4 self-assembles with guests into irreversible noncovalent complexes and what accelerates the assembly
Source: Commun Chem. 2022 Jan 20;5:9. doi: 10.1038/s42004-022-00624-4 (PMC9814096; doi:10.1038/s42004-022-00624-4)
Supplement: Supplementary file 3 — Supplemental material [file 42004_2022_624_MOESM3_ESM.pdf]

Supplementary Information

How neocarcerand Octacid4 self-assembles with guests into irreversible noncovalent complexes and what accelerates the assembly

Yuan-Ping Pang
E-mail: camdl1@icloud.com

Fig. S1……………………………………………………………………………………Pages 2
Tables S1 to S3………………………………………………………………………………Pages 3–6
Supplementary Note 1…………………………………………………………………………Page 7

Fig. S1. Chemical structures of HC2 and HCD2 and the assembly of HC2 into the neutral Octacid4. Thick lines indicate the bonds between main-chain atoms. Thin lines indicate the bonds between side-chain atoms or between a main-chain atom and a side-chain atom. Thin dashed lines and colored lines indicate the respective intra- and inter-residue bonds that are constructed with cross-links. The double bonds and net charges are not displayed for clarity.

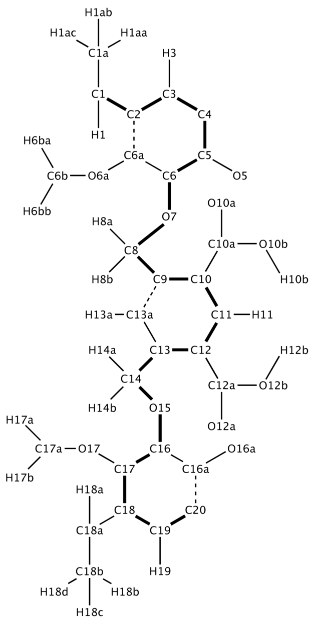

HC2

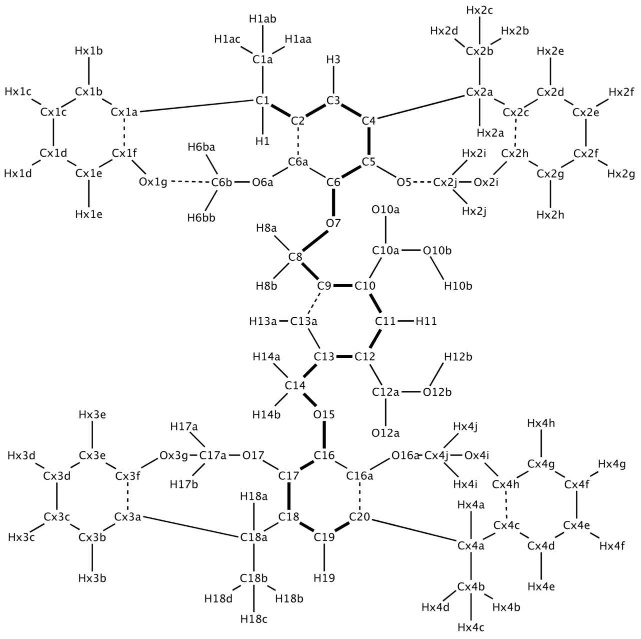

HCD2

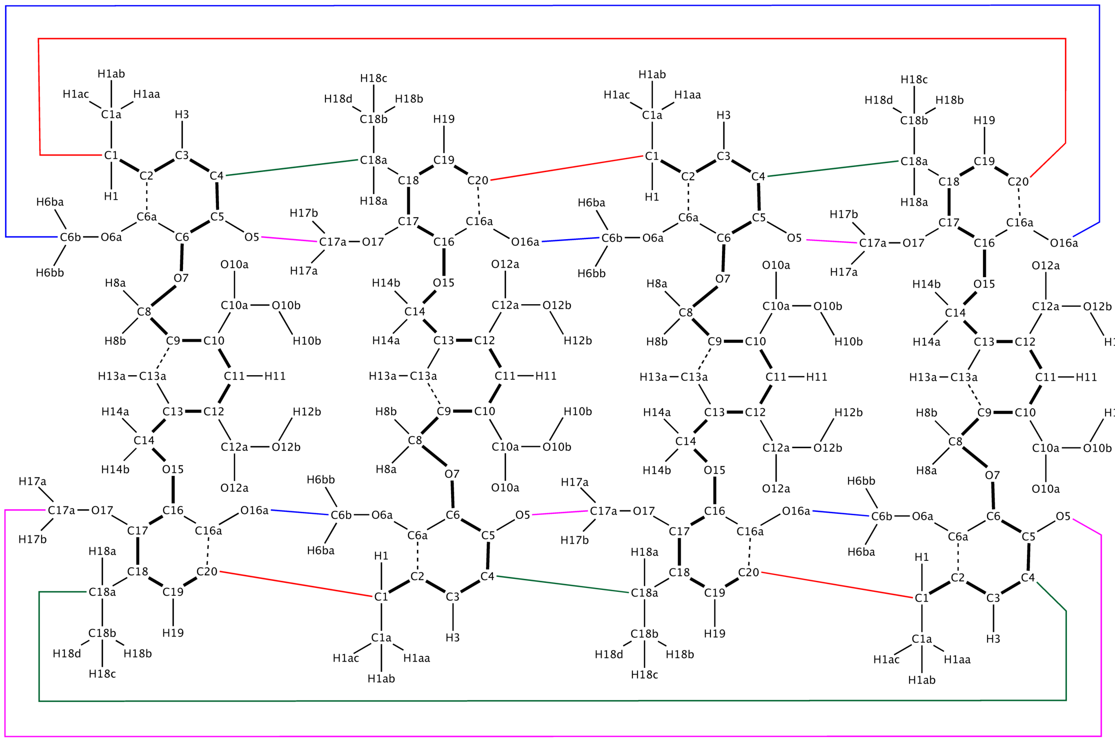

Octacid4 (in the neutral form)

Table S1. A complete list of systems simulated, their conditions and results.

Octacid4 configuration	Guest	Copies of guest	Copies of Na^+	Copies of NaCl	Solvent water	Temp (K)	Thermostat	Simulation Time (ns)	Number of simulations	Pathways captured
Octa-anionic, *apo*	xylene	10	8	60	2,359	298	Berendsen	14,252	20	0
Octa-anionic, *apo*	xylene	10	8	60	2,359	340	Berendsen	14,252	20	0
Octa-anionic, *apo*	xylene	10	8	60	2,359	363	Berendsen	14,252	20	0
Octa-anionic, *apo*	xylene	10	8	60	2,359	370	Berendsen	14,252	20	0
Neutral, *apo*	xylene	150	0	0	0	298	Berendsen	14,252	20	0
Octa-anionic, $5H_2O$	xylene	150	8	0	0	298	Berendsen	316	40	25
Octa-anionic, *apo*	xylene	250	8	0	0	298	Berendsen	316	40	10
Octa-anionic, *apo*	xylene	250	8	0	0	298	Berendsen	632	40	12
Octa-anionic, *apo*	xylene	150	8	0	0	298	Langevin	632	50	6
Octa-anionic, *apo*	xylene	150	8	0	0	298	Berendsen	14,252	40	40
Octa-anionic, *apo*	dioxane	150	8	0	0	298	Berendsen	6,320	100	2
Octa-anionic, *apo*	dioxane	150	8	0	0	340	Berendsen	12,640	40	24
Octa-anionic, *apo*	naphthalene	150	8	0	0	298	Berendsen	6,320	100	1
Octa-anionic, *apo*	naphthalene	150	8	0	0	340	Berendsen	6,320	100	4
Octa-anionic, *apo*	naphthalene	150	8	0	0	363	Berendsen	7,900	100	10

Table S2. Individual priming, ingression, and complexation times and linker configurations.

Simulation ID	Priming (ns) HH/COM Cutoff	Ingression (ps) HH/COM Cutoff	Complexation (ns) HH/COM Cutoff	Configuration	bidendate linkers	Cation-pi	Na chelation
p-Xylene at 298 K (HH distance cutoff = 2.6 Å and COM distance cutoff = 10 Å)							
1	1261/1261	174/179	1261/1261	coplanar	1	yes	no
2	299/299	53/53	299/299	orthogonal	0	no	no
3	2488/2488	934/934	2489/2489	coplanar	2	no	no
4	369/369	13/10	369/369	parallel	1	yes	no
5	26/26	13/13	26/26	orthogonal	0	yes	no
6	287/287	22/23	287/287	orthogonal	0	no	no
7	98/98	43/51	98/98	parallel	0	yes	no
8	246/246	16/41	246/246	orthogonal	0	yes	no
9	772/772	8/8	772/772	parallel	0	yes	no
10	528/528	48/48	528/528	parallel	1	no	no
11	296/296	141/143	296/296	parallel	0	yes	no
12	462/462	8/8	462/462	orthogonal	0	no	no
13	14/14	100/214	14/14	orthogonal	0	no	no
14	53/53	829/829	53/53	orthogonal	0	yes	no
15	203/203	61/66	203/203	parallel	0	yes	no
16	14199/14199	291/289	14200/14200	coplanar	2	yes	no
17	14/14	9/46	14/14	orthogonal	0	no	no
18	237/237	13/61	237/237	orthogonal	0	no	no
19	12/12	21/62	12/12	orthogonal	0	no	no
20	141/141	48/48	141/141	orthogonal	0	no	no
21	41/41	40/45	41/41	orthogonal	0	no	no
22	3049/3049	42/55	3049/3049	orthogonal	1	yes	no
23	58/58	83/83	58/58	orthogonal	0	yes	no
24	636/636	8/7	636/636	parallel	1	yes	no
25	1390/1390	53/69	1390/1390	orthogonal	0	no	no
26	1476/1476	28/28	1476/1476	orthogonal	1	yes	no
27	670/670	107/109	670/670	coplanar	0	no	no
28	1032/1032	33/33	1032/1032	parallel	1	yes	no
29	1504/1504	45/45	1504/1504	coplanar	0	no	no
30	341/341	63/63	341/341	parallel	1	no	no
31	171/171	64/64	171/171	orthogonal	0	no	no
32	162/162	101/101	162/162	orthogonal	0	yes	no
33	1660/1660	16/87	1660/1660	coplanar	2	no	no
34	320/320	42/42	320/320	parallel	1	yes	no
35	83/83	90/91	83/83	orthogonal	0	yes	no
36	458/459	24/22	459/459	coplanar	0	no	no
37	675/675	52/52	675/675	parallel	0	yes	no
38	2728/2728	24/25	2728/2728	parallel	1	no	no
39	1287/1287	12/12	1288/1288	orthogonal	0	yes	no
40	1117/1117	33/39	1117/1117	orthogonal	0	no	no

1,4-dioxane at 298 K (HH distance cutoff = 2.6 Å and COM distance cutoff = 8 Å)							
30	3668/3668	6/6	3668/3668	parallel	1	no	no
76	5681/5681	52/52	5681/5681	parallel	0	no	yes
1,4-dioxane at 340 K (HH distance cutoff = 2.6 Å and COM distance cutoff = 8 Å)							
1	798/798	13/6	798/798	parallel	0	no	no
3	471/471	50/50	471/471	orthogonal	1	no	no
5	511/511	26/26	511/511	parallel	0	no	no
6	492/492	8/8	492/492	orthogonal	0	no	no
7	1814/1814	17/17	1814/1814	parallel	0	no	no
8	567/567	21/21	567/567	parallel	1	no	no
10	87/87	9/9	87/87	parallel	0	no	no
14	1285/1285	33/33	1285/1285	parallel	0	no	no
16	63/63	19/14	63/63	parallel	0	no	yes
17	327/327	17/17	327/327	orthogonal	0	no	no
22	335/335	22/22	335/335	parallel	0	no	yes
23	920/920	20/7	920/920	parallel	0	no	no
24	466/466	14/14	466/466	parallel	0	no	no
25	34/34	29/29	34/34	parallel	0	no	no
27	963/963	19/19	963/963	parallel	0	no	no
29	112/112	8/8	112/112	parallel	0	no	no
32	116/116	22/22	116/116	parallel	0	no	no
33	860/860	15/15	860/860	parallel	0	no	yes
34	2314/2314	8/8	2314/2314	parallel	0	no	no
35	2816/2816	6/6	2816/2816	parallel	0	no	no
36	329/329	26/26	329/329	parallel	0	no	no
37	1263/1263	6/6	1263/1263	parallel	0	no	no
38	254/254	13/13	254/254	parallel	0	no	no
40	945/945	9/5	945/945	parallel	0	no	no
Naphthalene at 298 K (HH distance cutoff = 2.6 Å and COM distance cutoff = 10 Å)							
34	5721/5721	88/88	5721/5721	parallel	0	no	no
Naphthalene at 340 K (HH distance cutoff = 2.6 Å and COM distance cutoff = 10 Å)							
9	1865/1865	257/257	1866/1866	parallel	1	yes	no
27	3531/3531	2189/2185	3533/3533	orthogonal	0	yes	no
70	4389/4389	16/14	4389/4389	parallel	1	yes	no
98	1904/1904	216/216	1905/1905	orthogonal	0	yes	no
Naphthalene at 363 K (HH distance cutoff = 2.6 Å and COM distance cutoff = 10 Å)							
2	7180/7180	100/100	7180/7180	coplanar	2	no	no
10	7234/7234	8892/8892	7243/7243	coplanar	2	no	no
31	4719/4719	63/59	4719/4719	coplanar	2	no	no
39	6123/6123	31035/31032	6154/6154	coplanar	2	no	no
46	5940/5940	27913/27912	5968/5968	coplanar	2	no	no
47	4870/4870	51/45	4870/4870	coplanar	2	no	no
61	4238/4238	55/66	4238/4238	coplanar	2	no	no
85	5305/5305	7663/7662	5312/5312	coplanar	2	no	no
87	1457/1457	1730/1730	1458/1458	orthogonal	1	yes	no
91	651/651	399/399	652/652	parallel	0	yes	no

Table S3. The C17a-C6b distances of the entrance and opposing portals.

Guest at 298 K	Entrance (Å)	Opposing (Å)
XYP	7.00	5.30
XYP	7.30	5.80
XYP	7.10	5.60
XYP	6.80	5.50
XYP	6.70	5.50
XYP	7.10	5.20
XYP	6.80	5.70
XYP	6.80	5.70
XYP	7.00	6.20
XYP	7.30	5.80
XYP	7.30	5.40
XYP	7.00	5.50
XYP	7.10	5.20
XYP	7.20	6.50
XYP	7.30	5.10
XYP	7.50	5.40
XYP	7.00	5.40
XYP	7.10	5.40
XYP	6.90	5.70
XYP	7.20	5.40
XYP	6.90	5.40
XYP	7.00	5.70
XYP	7.20	5.70
XYP	6.70	5.70
XYP	7.10	5.30
XYP	7.00	5.80
XYP	7.20	5.30
XYP	7.20	5.80
XYP	7.10	5.40
XYP	7.00	5.80
XYP	6.80	6.10
XYP	7.60	5.40
XYP	6.90	6.00
XYP	7.00	5.60
XYP	7.30	5.50
XYP	6.90	5.60
XYP	7.10	5.40
XYP	6.80	5.70
XYP	6.90	5.10
XYP	7.10	5.60
DIO	6.70	6.00
DIO	6.80	5.10
NAP	6.90	4.80
Avg	7.04	5.56
SD	0.21	0.32
SE	0.03	0.05
Lower 95%CI	6.98	5.46
Upper 95%CI	7.10	5.66

XYP: *p*-Xylene, DIO: 1,4-Dioxane, NAP: Naphthalene, Avg: Average, SD: Standard deviation, SE: standard error, Lower 95%CI: the lower bound of the 95% confidence interval, and Upper 95%CI: The upper bound of the 95% confidence interval.

Supplementary Note 1

Instruction on how to view a movie frame by frame on macOS using QuickTime Player and Windows using either Windows Media Player or VLC Media Player.

QuickTime Player: Open the movie in QuickTime Player, move the cursor to point to the small vertical progress bar and then press the left mouse button to drag the frame forward or backward, or simply press the right or left arrow key to forward or reverse the frame.

Windows Media Player: Open the movie in Windows Media Player, pause the movie, right click on the movie, click Enhancements, and then click Play Speed Settings. In the pop-up use the forward or backward arrow to view the next or previous frame.

VLC Media Player: Open the movie in VLC Media Player, click the left arrow to play and immediately pause the movie or pause before the frame of interest. Press the e key on the keyboard to view the next frame.
